# A high-fat, high-fructose diet induced hepatic steatosis, renal lesions, dyslipidemia, and hyperuricemia in non-obese rats

**DOI:** 10.1016/j.heliyon.2022.e10896

**Published:** 2022-10-03

**Authors:** Ika Yustisia, Delvina Tandiari, Muhammad Husni Cangara, Firdaus Hamid, Nu'man AS. Daud

**Affiliations:** a Master Program of Biomedical Science, Graduate School Hasanuddin University, Makassar, South Sulawesi, Indonesia; b Department of Biochemistry, Faculty of Medicine, Hasanuddin University, Makassar, South Sulawesi, Indonesia; c Department of Anatomic Pathology, Faculty of Medicine, Hasanuddin University, Makassar, South Sulawesi, Indonesia; d Department of Microbiology, Faculty of Medicine, Hasanuddin University, Makassar, Indonesia; e Department of Internal Medicine, Faculty of Medicine, Hasanuddin University, Makassar, Indonesia

**Keywords:** High-fat diet, High-fructose diet, Liver steatosis, Renal lesions, Non-obese rats

## Abstract

Excessive consumption of fat and sugar is associated with various chronic diseases. However, the variation of fat and sugar content in the diet greatly affected the outcome. In this study, a high-fat, high-fructose diet (HFHFD) formula was made with a composition of 31.99% carbohydrate, 40.7% fat, 11.8% protein, and an additional 30% fructose drink to confirm the effects of HFHFD on metabolic health and pathological changes in organs, especially the liver, kidneys, pancreas, muscles, and spleen. A total of 24 male Wistar rats aged 8–12 weeks were divided into four groups: standard chow (SC), HFHFD, SC + carbon tetrachloride (CCl4), and HFHFD + CCl4. After eight weeks of dietary intervention, body mass index, obesity index, lipid profiles, liver function tests, fasting blood glucose, serum uric acid and urea levels, and tissue histopathology were examined. HFHFD with the main unsaturated fatty acids of linoleic acid (14.57%) and palmitoleic acid (8.28%), the main saturated fatty acids of stearic acid (13.62%) and myristic acid (10.09%), and a low trans-fatty acids content, did not promote the rats to become obese. However, liver histology examination showed severe hepatic steatosis (78.33%), leading to steatohepatitis accompanied by an increase in serum ALP (p < 0.01), triglyceride (p < 0.001), total cholesterol (p < 0.05), and uric acid (p < 0.001) levels. Other histological features showed moderate lesions (45%) of the kidney, slight vacuolization of the pancreas, and a mild increase of inflammatory cells in the spleen and muscle. So, this study found that although HFHFD did not promote obesity within 8 weeks of administration, it induced hepatic and renal lesions, dyslipidemia, and hyperuricemia as a metabolic consequence of excessive fatty acids and fructose.

## 1 Introduction

Fats and sugars are the main energy source for cells to carry out their physiological functions, so they must be contained in food. However, foods high in fat and sugar generally taste good, so they are often consumed in excess. Global data showed that the consumption of fat and sugar in the world's population tends to increase from year to year [1]. Both macronutrients have a bad health impact if consumed in excess. Studies have shown that excessive fat and sugar consumption is a risk factor for chronic diseases such as metabolic syndrome, obesity, cardiovascular disease, type 2 diabetes mellitus, Alzheimer's disease, and non-alcoholic fatty liver disease (NAFLD) [2].

Many studies have been conducted to understand the mechanism of diseases due to excess fat and sugar intake using animal models, especially rodents [3]. However, some issues still require further explanation because several studies showed varying results due to differences in diet composition. Because the results of all the studies in this field should mainly be translated to conditions that occur in humans, the diet composition administered to animal models should be in accordance with the levels routinely consumed by humans [3, 4]. In addition, humans consume foods high in fat and sugar as the main meal and a side dish. Fats are generally obtained from oils, butter, coconut milk, and margarine. At the same time, sugars are obtained from staple foods such as rice, wheat, corn, millet, and sorghum, as well as added sugars such as those contained in various kinds of sweet drinks, so the most rational approach to studying the impact of excessive consumption of fat and sugar is through the provision of a diet that combines the two, such as a high-fat, high-carbohydrate diet with additional sugar components such as glucose, fructose, or sucrose.

The high-fat diets applied in various studies varied greatly depending on the composition of their constituent fatty acids [4]. These difference in fatty acid composition could affect the results of experiments with varying outcomes. For example, fats containing long-chain saturated fatty acids stimulated hepatic lipotoxicity [5] and could increase body weight more than trans fatty acids after 6–8 weeks of intervention [6, 7]. However, trans fatty acids induced more steatosis, poorer lipid profiles, and insulin resistance [6, 7]. Meanwhile, a high-fat diet containing medium-chain triglycerides (MCTs) did not induce weight gain but maintained metabolic health and induced hepatic thermogenesis [8]. Even in obese conditions, a diet rich in MCTs could promote body fat depletion and improved metabolic health [8]. Another study showed monounsaturated fatty acids to have a protective effect against palmitic saturated fatty acid-induced lipotoxicity [9]. Therefore, high-fat diets could be categorized into obesogenic and non-obesogenic HFD [2]. Furthermore, clinical studies revealed that NAFLD in lean individuals had a reasonably high prevalence (16%), suggesting that lipid toxicity was not always preceded by excess weight gain or obesity [10, 11]. Therefore, more studies are needed on variations in high-fat diets to clarify the mechanism and course of metabolic disorders or organ toxicity in the body due to exposure to excess fat.

In contrast to HFD, a high-sugar diet (HSD) has a tissue-damaging impact without significantly expanding fat mass. Studies have shown that a diet with 30% of its energy from fructose within 2 weeks could induce mitochondrial changes in the rat brain [12]. Another study with 10% g/vol fructose for 12 weeks decreased antioxidant enzymes, superoxide dismutase 1 (SOD1), and catalase in the cortex and striatum and was associated with increased hyperactivity behavior in mice [13]. Because excess sugars are the primary source of lipogenesis, like HFD, HSD could be obesogenic if given for a long time and induced mitochondrial dysfunction and oxidative stress that underlay tissue damage and decreased organ function [14]. In addition, another study stated that HSD, especially a high-sucrose diet, induced more significant and faster metabolic changes than a high-fat diet after 8 months of intervention [15]. Because of the different effects of HFD and HSD when administered separately, more studies are needed to combine these two diets to demonstrate their simultaneous effects on metabolic health and organ function.

In this study, we formulated a high-fat diet from corn, bran, shrimp shell powder, coconut meal, beef tallow, coconut oil, wheat flour, and soybean, with an expected fat content above 40% combined with a high-fructose drink. The purpose of this study was to augment scientific evidence of the impact of HFHFD on metabolic health and pathological changes in organs, especially the liver, kidneys, pancreas, muscles, and spleen at 8 weeks of administration.

## 2 Materials and methods

### 2.1 Animals

The animal handling and experimentation protocol of this study has been approved by the Animal Ethics Committee from the Faculty of Medicine Ethics Commission, Hasanuddin University, with letter number 75/UN4.6.4.5.31/PP36/2021. Twenty-four male rats (Rattus norvegicus strain Wistar) were used. Animals were obtained from rat breeders for research purposes in Makassar City, South Sulawesi, Indonesia. Rats aged 8–12 weeks with weights ranging from 200-250 g were healthy and active. The rats were acclimatized for one week and then transferred to each cage containing one rat with 12 h of light and 12 h of darkness; room temperature ranged from 26-28 °C, with humidity 50% ± 10%. Rats were randomly divided into four groups where each group consisted of six rats: 1) SC group was fed standard rodent chow; 2) SC + CCl4 group was fed standard rodent chow and injected intraperitoneally with 0.08 mL/kg of body weight CCl4; 3) HFHFD group was fed a high-fat and high-fructose diet; 4) HFHFD + CCl4 group was fed a high-fat and high-fructose diet and injected intraperitoneally with 0.08 mL/kg of body weight CCl4.

### 2.2 Dietary and CCl4 treatment

The standard rodent chow that was given to groups 1 and 2 was factory-made (Van der voer, Indonesia) with the raw materials as stated on the label: soybean meal, bran, palm meal, corn, palm oil, premises, soluble dried grain, wheat pollard; with the nutritional content of 20% protein, 37% carbohydrates, 7% fat, 15.9% fiber, 0.8% phosphorus, and 1% calcium with a total calorie of 2.91 kCal/gr. High-fat diet (HFD) given to groups 3 and 4 was made from corn, bran, shrimp shell powder, coconut meal, beef tallow, coconut oil, wheat flour, and soybean meal using a formulation based on Nutrisurvey software. Test results conducted at the Laboratory of Animal Food Chemistry, Faculty of Animal Husbandry, Hasanuddin University showed the nutritional content of HDF: 11.8% protein, 31.99% carbohydrates, 40.7% fat, 4.5% fiber, 0.63% phosphorus, 0.38% calcium with a total calorie of 5.45 kCal/gr. Cholesterol and fatty acid content of both types of diet were examined using the Soxhlet extraction method and analyzed using the Gas Chromatography-Flame Ionization Detector (GC-FID) technique. All rats received 25–30 g per day of diet according to their group using formula 10% of the rat's body weight for eight weeks and were weighed every week. Groups 1 and 2 received drinking water ad libitum. Groups 3 and 4 received 40 mL fructose water daily containing high fructose syrup (Rose Brand, Indonesia) and water with a ratio of 3:7 (final concentration: 0.2 gr/mL) so that these groups received an additional calorie of 4.38 kCal/gr.

Groups 2 and 4 received a low dose of CCl4 via intraperitoneal injections twice weekly, according to the protocol used by the previous study [16]. CCl4 solution for analysis (≥99.5%; Merck; 1.02222.2500) was diluted using corn oil at a final concentration of 5 μL/mL and injected at a dose of 0.08 mL/kg of body weight.

The study lasted for eight weeks. On the final day of the experiment, the rats' body weight and length were measured, and feeding was stopped 12 h before blood collection. The obesity index in rats was determined using the Lee index with the formula: body weight (g)^1/3^/naso-anal length (cm). The rats were indicated as obese if they had a Lee index of over 0.3 [17,18]. Body mass index (BMI) measurements were also carried out using the formula: body weight (g)/naso-anal length^2^ (cm^2^), where BMI for male adult Wistar rats ranged between 0.45 and 0.68 g/cm^2^ [19]. Approximately 2 mL of blood was sampled from the lateral tail vein. Blood was placed into a sterile tube and then centrifuged using a refrigerated centrifuge (4 °C) at a speed of 2000 x g to obtain approximately 1 mL of serum. The liver, pancreas, kidneys, spleen, and thigh muscles were harvested and processed for histological examination.

### 2.3 Biochemical parameters

The level serum of lipid profiles [triglycerides (TG), total cholesterol, and HDL-cholesterol], uric acids, urea, liver function tests [alanine aminotransferase (ALT), aspartate aminotransferase (AST), and alkaline phosphatase (ALP)], and fasting blood glucose (FBG) were examined. Serum TG, urea, total cholesterol, and uric acid levels were determined by enzymatic hydrolysis according to the protocol of a commercial kit, namely Quimica Clinica Aplicada, Spain, for TG and urea [20, 21]. ReiGed Diagnostics, Turkey, for total cholesterol and uric acid [22, 23]. ALP was measured kinetically based on the kit instructions by Glory Diagnostics, Spain [20]. The absorbance of these biochemical parameters was measured using Genesys 150 UV VIS from Thermo Scientific. ALT and AST examinations were measured using a test kit from Mindray chemistry reagent and measured with a Mindray BS 220E Chemistry Analyzer, China. FBG and HDL-cholesterol were measured using the point of care test, GlucoDr®, and LipidPro®, respectively.

### 2.4 Histological examination

Sliced organs of the liver, pancreas, kidney, spleen, and biceps femoris were processed into formalin-fixed, paraffin-embedded sections and stained with hematoxylin and eosin (HE). The tissue steatosis score was evaluated by an expert pathologist and performed blinded, without knowing what treatment was given to each tissue preparation. The scoring system for each organ histology was performed according to protocols from previous studies, as summarized in Table 1.

**Table 1:** Scoring system for organ histology examination.

Organs	Scoring system or descriptive evaluation	References
Liver	Liver steatosis is determined by the presence of fat accumulation in hepatocytes in the form of microvesicular/macrovesicular steatosis with criteria: grade 0 (normal, <5%); grade 1 (mild, 5%–33%); grade 2 (moderate, 34–66%), and grade 3 (severe, >66%).	[24, 25]
Pancreas	Pancreas histopathology was determined based on the presence of vacuolization, fatty infiltration, relative number of islets, islet deformations, and hemosiderin content; with a score of 0 = none, 1 = mild; 2 = moderate; and 3 = weight.	[26]
Kidney	Renal histopathology was determined based on the presence of hydrophilic degeneration, hemorrhagic, inflammation, glomerular capillary proliferation, with a score of 0 = no lesion; 1 = minimum, < 10%; 2 = mild; 10–25%; 3 = moderate, 26%–50%; 4 = severe; > 50%	[27] (with modification)
Spleen	Descriptive assessment based on: 100x magnification A. Billroth cord thickened B. Congestion C. Accumulation of hemosiderin + fat in the pulp of rubra 400x magnification 1. Macrophages with hemosiderin 2. Necrotic Lymphocytes 3. Intercellular hemosiderin 4. Lymphocytes +	[28] (with modification)
Biceps femoris	The count of myocytes and inflammatory cells in three microscope fields of view.	-

### 2.5 Statistical analysis

Data with statistical tests are presented in the mean ± standard deviation (SD). The test used was one-way ANOVA followed by Tukey HSD (honestly significant difference). Student t-test was used to compare the mean value of the number of inflammatory cells in muscle tissues between the SC and HFHFD groups. The statistical tests were carried out using Microsoft Excel and an online calculator available at https://www.socscistatistics.com/tests/anova/default2.aspx.

## 3 Results

### 3.1 Cholesterol and fatty acids content in standard chow and HFD

The cholesterol and fatty acid content of the standard chow (Van der voer) and HFD are shown in Table 2. HFD contained total cholesterol (3.12%) higher than that of standard chow (2.09%). The two types of diets had different fatty acid content. Of the 19 fatty acids detected in the standard chow, 68.4% and 31.6% were saturated fatty acids and unsaturated fatty acids, respectively, with linolelaidic acid (polyunsaturated trans fatty acid), palmitic acid, stearic acid, lauric, and caproic acid as the five fatty acids with the highest percentage. HFD contains 58.8% saturated and 42.2% unsaturated fatty acids of the 17 fatty acids detected, with linoleic acid, stearic acid, myristic acid, palmitoleic acid, and pentadecanoic acid as the five fatty acids with the highest percentage. Only stearic acid has almost the same percentage in both types of diet (12.41% vs. 13.62% for SC vs. HFD).

**Tabel 2:** Cholesterol and fatty acids contained in standard chow and HFD.

No.	Cholesterol and fatty acids	Standard chow (Van der voer) (%)	HFD (%)
1	Cholesterol	2.09	3.12
2	Caproic acid (C6:0)	3.16	0.04
3	Caprylic acid (C8:0)	1.4	0.06
4	Undecanoic acid (C11:0)	ND	0.17
5	Capric acid (C10:0)	0.29	ND
6	Lauric acid (C12:0)	3.53	0.54
7	Tridecanoic acid (C13:0)	ND	0.08
8	Myristic acid (C14:0)	3.01	10.09
9	Pentadecanoic acid (C15:0)	0.22	3.63
10	Palmitic acid (C16:0)	17.11	1.37
11	Palmitoleic acid (C16:1)	1.05	8.28
12	Heptadecanoic acid (C17:0)	0.23	2.33
13	Stearic acid (C18:0)	12.41	13.62
14	Elaidic acid (C18:1n9t)	0.79	0.09
15	Linoleic acid (C18:2n6c)	0.23	14.57
16	Linolenic acid (C18:3n3)	0.82	ND
17	Oleic acid C18:1n9c	ND	0.97
18	Linolelaidic acid (C18:2n9t)	39.35	0.99
19	Arachidic acid (C20:0)	1.78	1.39
20	Behenic acid (C22:0)	0.78	ND
21	Tricosanoic acid (C23:0)	0.14	ND
22	Lignoceric acid (C24:0)	1.18	ND
23	Cis-5,8,11,14,17-eicosapentaenoic acid (C20:5n3)	0.77	0.31

### 3.2 Daily food and water intake

In the first week of the experiment, rats fed a high-fat diet, HFHFD and HFHFD + CCl4, consumed less feed (8.64 ± 0.50 g and 7.69 ± 0.71 g, respectively) than the group fed the standard diet, SC and SC + CCl4 (19.67 ± 1.02 g and 18.79 ± 1.25 g, respectively). If converted into calorie units, the average caloric intake in the first week for SC, SC + CCl4, HFHFD, and HFHFD + CCl4 were 57.23 kCal, 60.74 kCal, 54.67 kCal, and 56.47 kCal, respectively. These results indicated that in the first week, the four experimental groups had no difference in calorie intake. However, when entering the second week, there was an increase in food intake by the HFD groups, which persisted until the end of the study. Thus, as shown in Table 3, the HFD groups consumed almost the same weight as the standard diet group but had significantly higher calorie intake (p < 0.001) due to the high-fat and fructose content of the HFHFD. There was no significant difference in food intake between HFHFD vs. HFHFD + CCl4 as well as SC vs. SC + CCl4. For water intake, the HFHFD and HFHFD + CCl4 groups consumed less drinking water than the SC and SC + CCl4 groups.

**Table 3:** Mean daily feed intake and water intake of the four experimental groups.

Intake		SC	HFHFD	SC + CCl4	HFHFD + CCl4
Feed intake	gr	22.4 ± 0.21	22.3 ± 0.71	21.8 ± 0.23	22.2 ± 0.75
	kCal	65.15 ± 0.618	136.20 ± 3.904^a^	63.34 ± 0.667	135.80 ± 4.117^b^
Water intake (mL)		23.30 ± 0.274	16.59 ± 0.201	23.00 ± 0.220	16.65 ± 0.272

Values are means ± SD. Significant values were based on the one-way ANOVA test, followed by the post-hoc Tukey's HSD.

SC, standard chow; HFHFD, high-fat high-fructose diet; CCl4, carbon tetrachloride.

a) p < 0.001 HFHFD vs SC and SC + CCl4; b) p < 0.001 HFHFD + CCl4 vs SC and SC + CCl4.

### 3.3 Body weight, obesity index, and BMI

**Figure 1:**
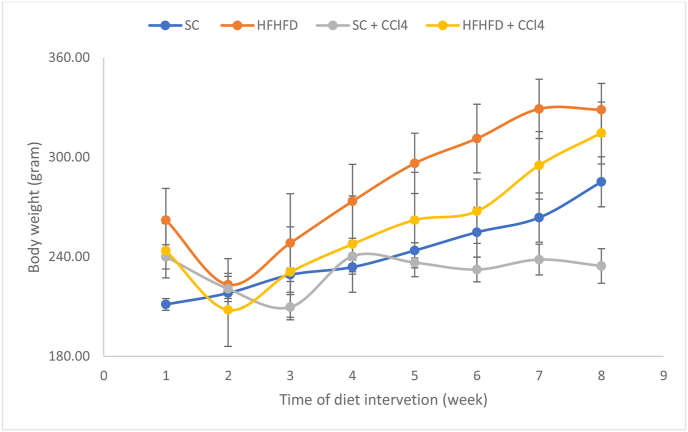
Changes of rats' body weight during an 8-week diet intervention.

Entering the second week of the experiment, the body weight of rats (Figure 1) in the SC, HFHFD, and HFHFD + CCl4 groups experienced a gradual increase but did not show significant differences between groups. After entering the week 6, there was a significant difference in body weight gain, with the highest order being the HFHFD group followed by HFHFD + CCl4, and SC, and this pattern persisted until the end of the study. Meanwhile, the body weight of rats in SC + CCl4 group were fluctuated and then settled in the range of 235.42 ± 2.58 g in the week 5 until the end of the experiment. For the weight gain (initial body weight subtracted by final body weight in Table 4), the SC group showed the highest weight gain, followed by HFHFD + CCl4 and then HFHFD, while the SC + CCl4 group experienced weight loss. The Lee index used to determine obesity in rats showed that the four groups were not significantly different (p = 0.048), although the HFHFD and HFHFD + CCl4 groups had indices slightly above 0.3 (Table 4) as the limit value for determining obesity. Furthermore, BMI showed that the HFHFD group was significantly higher than SC and SC + CCl4, and HFHFD + CCl4 was significantly higher than SC + CCl4 but not SC. However, the BMI of these four groups was still in the normal range of BMI of rats, namely 0.45–0.68 g/cm^2^.

**Table 4:** Body weight, obesity index, and BMI after 8 weeks of dietary interventions.

Measurements	Groups			
	SC	HFHFD	SC + CCl4	HFHFD + CCl4
Initial weight (g)	211.33 ± 8.68	262.17 ± 47.87	240.00 ± 17.81	243.67 ± 40.07
Final weight (g)	285.17 ± 36.89	328.67 ± 38.98	234.50 ± 25.52	314.67 ± 45.66
Weight gain (g)	87 ± 13.21^a^	66.5 ± 35.10^a^	-5.5 ± 15.13	71.00 ± 33.36^a^
Naso-anal length (cm)	21.58 ± 1.43	22.9 ± 1.25	20.92 ± 0.38	22.2 ± 1.14
Lee index (gr/cm)	0.30 ± 0.01	0.31 ± 0.01	0.29 ± 0.01	0.31 ± 0.01
BMI (g/cm^2^)	0.57 ± 0.04	0.66 ± 0.05^b^	0.54 ± 0.05	0.65 ± 0.05^c^

Values are means ± SD. Significant values were based on the one-way ANOVA test, followed by the post-hoc Tukey's HSD.

SC, standard chow; HFHFD, high-fat high-fructose diet; CCl4, carbon tetrachloride.

a) p < 0.01 (SC, HFHFD, HFHFD + CCl4) vs SC + CCl4; b) p < 0.05 HFHFD vs SC; p < 0.01 HFHFD vs SC + CCl4; c) p < 0.01 HFHFD + CCl4 vs SC + CCl4.

### 3.4 Liver steatosis

**Figure 2:**
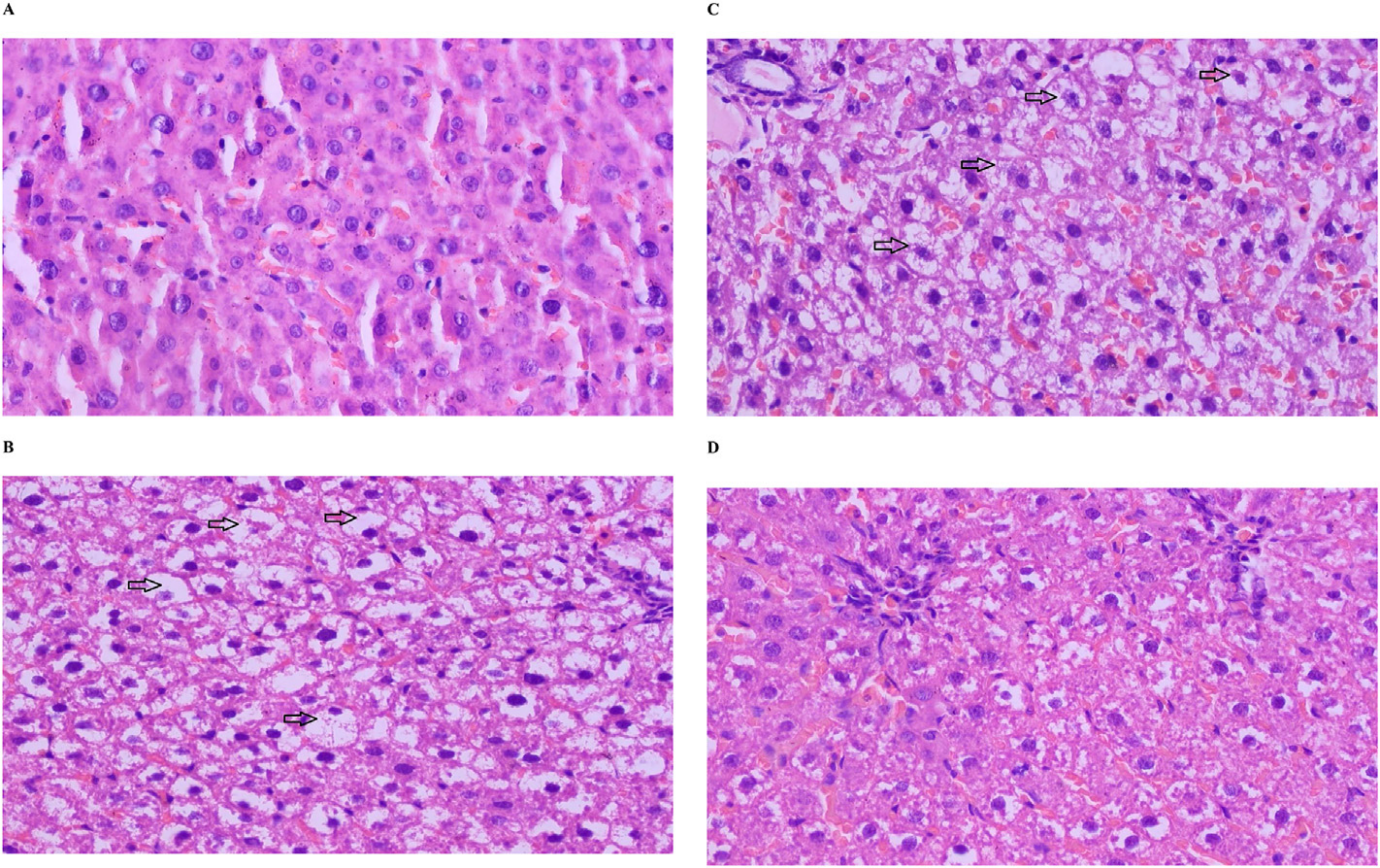
Representation of liver histology after 8 weeks of diet intervention (A) Normal liver histology of rat in SC group; (B) (C) (D) Liver histopathology of rats in HFHFD, HFHFD + CCl4, and SC + CCl4 group, respectively, which showed 100% (Grade 3), 80% (Grade 3), and 40% (Grade 2) of steatosis. The black arrow indicates feathery degenerations.

After 8 weeks of experimentation, the liver histology of rats (Figure 2 A-D and Table 5) in the HFHFD group (Figure 2 B) showed grade 3 steatosis with an average fat accumulation percentage of 78.33%. Rats in the HFHFD + CCl4 group (Figure 2 C) also showed grade 3 steatosis with a higher average percentage than HFHFD (88.33%). Rats in the SC + CCl4 group (Figure 2 D) had grade 1 to 2 steatosis with an average percentage of 13.33%. The livers of rats in the SC group (Figure 2 A) showed normal histology.

**Tabel 5:** Grading of liver steatosis after dietary intervention and micro-dose CCl4 administration.

Groups	Mean percentage of steatosis (%)	Grade of steatosis
SC	0.33	Grade 0
HFHFD	78.33	Grade 3
HFHFD + CCl4	88.33	Grade 3
SC + CCl4	13.33	Grade 1

### 3.5 Biochemical parameters after HFHFD and CCl4 micro-dose treatments

Biochemical parameters after 8 weeks of dietary interventions are shown in Table 6. ALP, TG, total cholesterol, and uric acid were the parameters most affected by HFHFD. The HFHFD, HFHFD + CCl4, and SC + CCl4 groups experienced an increase in ALP serum levels of 4.51, 4.89, and 2.10 times, respectively, compared to the SC group. For serum TG levels, the HFHFD and HFHFD + CCl4 groups experienced an increase of 2.45 and 2.14 times compared to the SC group. Serum TG levels in the SC and SC + CCl4 groups were not significantly different. The highest serum total cholesterol level was in the HFHFD group, followed by HFHFD + CCl4, significantly higher than in the SC and SC + CCl4 groups. Serum uric acid levels showed results that were almost in line with serum TG levels, where the HFHFD + CCl4 and HFHFD groups had an increase of 3.46 and 2.87 times, respectively, compared to the SC group, and the serum of the SC and SC + CCl4 groups did not differ significantly. Serum ALT, AST, HDL-cholesterol, glucose, and urea levels did not significantly differ between all groups. There was no significant difference in levels between the HFHFD and HFHFD + CCl4 groups in all biochemical parameters.

**Table 6:** Biochemical parameters after 8 weeks of dietary interventions.

Parameters	Groups			
	SC	HFHFD	SC + CCl4	HFHFD + CCl4
ALT (U/L)	42.60 ± 6.27	38.6 ± 2.61	38.80 ± 3.77	47.60 ± 7.23
AST (U/L)	148.40 ± 27.93	131.00 ± 7.35	129.60 ± 18.99	129.00 ± 6.32
ALP (U/L)	178.11 ± 30.34	803.90 ± 62.56^a^	373.15 ± 100.13	870.47 ± 228.43^b^
FBG (mg/dL)	243.40 ± 34.40	236.20 ± 13.87	239.50 ± 20.79	242.88 ± 9.77
Triglycerides (mg/dL)	105.18 ± 9.68	257.80 ± 74.81^c^	115.2 ± 8.32	224.6 ± 24.61^d^
Total cholesterol (mg/dL)	139.66 ± 13.79	180.74 ± 35.73^e^	133.32 ± 3.49	158.37 ± 22.27
HDL-cholesterol (mg/dL)	59.40 ± 11.72	59.80 ± 8.04	54.00 ± 3.16	61.80 ± 5.50
Uric acids (mg/dL)	2.54 ± 0.71	7.30 ± 0.59^f^	2.20 ± 0.16	8.78 ± 4.42^g^
Urea (mg/dL)	19.08 ± 3.89	16.76 ± 0.88	17.27 ± 4.06	15.61 ± 1.56

Values are means ± SD. Significant values were based on the one-way ANOVA test, followed by the post-hoc Tukey's HSD.

a) p < 0.01 HFHFD vs SC; HFHFD vs SC + CCl4; b) p < 0.001 HFHFD + CCl4 vs SC; HFHFD + CCl4 vs SC + CCl4; c) p < 0.001 HFHFD vs SC; HFHFD vs SC + CCl4; d) p < 0.01 HFHFD + CCl4 vs SC; HFHFD + CCl4 vs SC + CCl4; e) p < 0.05 HFHFD vs SC; HFHFD vs SC + CCl4; f) p < 0.001 HFHFD vs SC; HFHFD vs SC + CCl4; g) p < 0.001 HFHFD + CCl4 vs SC; HFHFD + CCl4 vs SC + CCl4.

### 3.6 Histological findings of pancreas, kidney, spleen, and muscle

**Figure 3:**
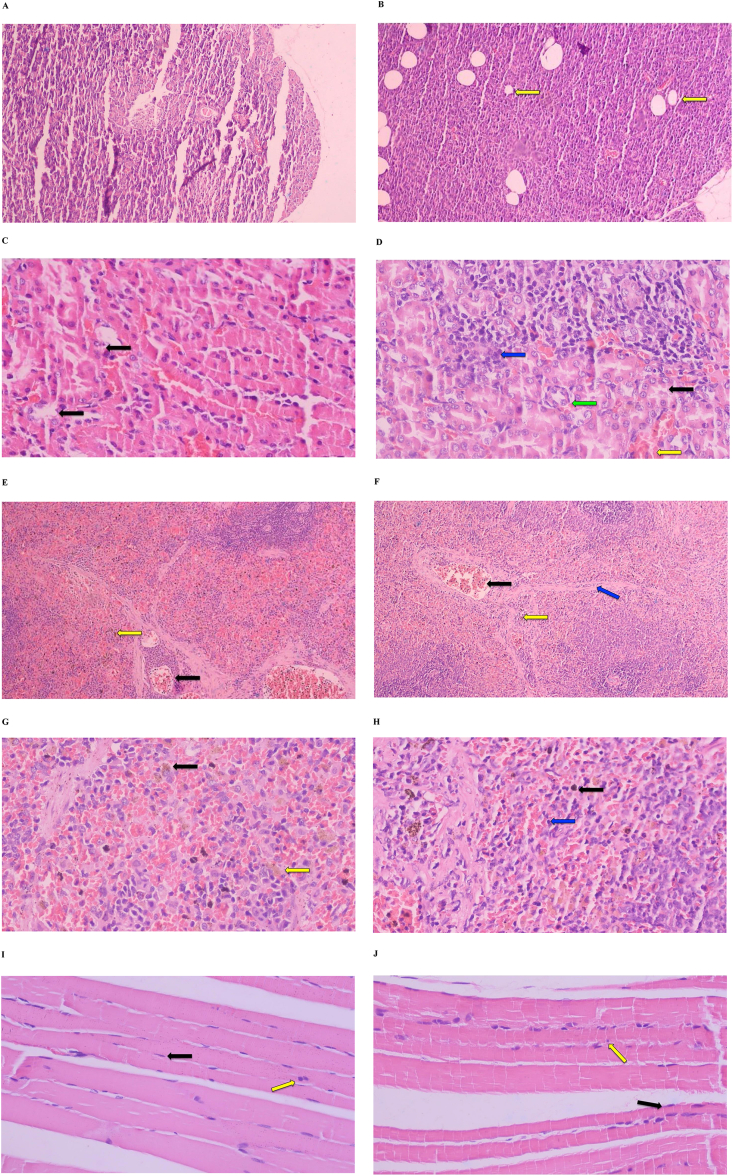
Histological features of the pancreas, kidney, spleen, and muscle. Representation of pancreas histology of SC rats (A) and HFHFD rats (B): islet deformation (black arrow), vacuolization (yellow arrow). Representation of kidney histology of SC rats (C) and HFHFD rats (D): hydrophilic degeneration (black arrow), hemorrhagic (yellow arrow), inflammation cells/lymphocytes (blue arrow), glomerular capillary (green arrow). Representation of 100x magnification spleen histology of SC rats (E) and HFHFD rats (F): congestion (black arrow), hemosiderin accumulation (yellow arrow), thickening of Billroth cord (blue arrow); 400x magnification spleen histology of SC rats (G) and HFHFD rats (H): hemosiderin laden macrophages (black arrow), intercellular hemosiderin (yellow arrow), lymphocytes (blue arrow). Representation of muscle histology of SC rats (I) and HFHFD rats (J): myocytes (black arrow), lymphocytes (yellow arrow).

Histological examination of the kidneys, pancreas, spleen, and muscle (Figure 3 A-J) was only performed on tissue sections from the HFHFD and SC groups to examine the effect of a high-fat and high-fructose diet on the histological changes of the four organs. The HFHFD group showed moderate kidney lesions (45%) in the form of hydropic degeneration, hemorrhagic, signs of inflammation, and glomerular capillary proliferation. While the SC group only showed mild lesions (7%) (Figure 3 C,D). Pancreatic tissues of the HFHFD group showed a slight vacuolization while the SC group appeared normal (Figure 3 A,B). The spleen tissues of the HFHFD group showed thickening of the Billroth cord with a higher number of lymphocytes per field of view than the SC group (Figure 3 E-H). The muscle tissue of the HFHFD group showed a significant increase in inflammatory cells (p = 0.013) compared to the SC group (Figure 3I,J).

## 4 Discussion

Many studies proved that the consumption of excess fats and carbohydrates underlay the occurrence of various chronic diseases. However, subsequent studies showed that not all fats and carbohydrates harm health. Medium-chain saturated and monounsaturated fats are referred to as “good fats” because they are less likely to promote insulin resistance, inflammation, or be stored as body fat compared to “bad fats,” long-chain saturated fats [4, 5, 6, 7, 8, 9, 29]. Analysis of lipid content in this present studied HFD (Table 2) showed slightly higher cholesterol levels (0.5x) with lower saturated fatty acid and higher unsaturated fatty acid content (linoleic acid and palmitoleic acid) compared to the standard diet. In addition, our high-fat diet contained minimal trans fatty acids compared to standard chow. However, this high-fat diet contained low medium-chain fatty acids with nearly the same content of long-chain saturated fatty acids (stearic acid) as the standard chow. Such feed composition did not appear to affect the appetite of the experimental animals, as indicated by almost equal food intake in all test groups, so the high-fat diet groups received higher calories than the standard chow groups (Table 3). The high sugar content in the drinking water of the HFHFD group seemed to affect the experimental animals' drinking behavior, which was characterized by a lower volume of water intake than the SC group.

The HFHFD formula applied in this study seemed insufficient to develop obesity in 8 weeks (Table 4). Although the BMI in the HFHFD group was significantly higher than in the SC group, it was still in the normal weight range of Wistar rats, 0.45–0.68 g/cm^2^ [19]. This result was supported by the Lee index, which is not significantly different between the four test groups. Indeed, in terms of weight gain, the SC group experienced the highest increase compared to the HFHFD group. These findings indicated that although the HFHFD group consumed higher calories than the SC group, the excess calories were not stored massively in fat, so the rats gained weight but did not achieve obesity within 8 weeks. Previous studies have found that the accumulation of body fats, which is a key condition in obesity, was strongly influenced by the fatty acid composition of the diet [30]. Long-chain saturated fatty acids tend to be stored in adipose tissue rather than being oxidized to form energy [29, 30, 31, 32]. Meanwhile, unsaturated fatty acids such as alpha-linolenic acid (ALA), oleic acid, and linoleic acid have a high oxidation rate compared to long-chain fatty acids such as palmitic and stearic acids [31, 32]. In addition, unsaturated fatty acids increased the thermogenic effect and oxygen consumption, indicating an increase in energy expenditure [33, 34]. Although the groups receiving CCl4 were not significantly different in food intake from those without CCl4, this was not the case in body weight changes. Rats on a standard diet but receiving a low dose of CCl4 did not gain weight with the lowest BMI and Lee's index. However, in the HFHFD + CCl4 group, food intake, weight gain, and Lee index were not significantly different compared with SC and HFHFD group. Further research is needed on the protective effect of a high-fat diet containing long-chain unsaturated fatty acids, such as linoleic acids and palmitoleic acids, against the toxic effects caused by CCl4.

Although the increase in body weight between the groups fed the standard diet versus the HFHFD was not significantly different, the fat accumulation in the liver was very different (Figure 2 A-D and Table 5). The HFHFD group clearly had fat accumulation in the liver up to 80% (Figure 2 B), while the SC group showed normal liver histology (Figure 2 A). The administration of CCl4 as a fatty liver accelerator [16] seemed to have a slight effect characterized by a percentage of steatosis in SC + CCl4 (Figure 2 D) vs. SC (Figure 2 A) and HFHFD + CCl4 (Figure 2 C) vs. HFHFD (Figure 2 B) only higher around 10%–13%. Thus, it seemed that HFHFD alone was adequate to induce severe hepatic steatosis. These results were in line with previous studies, which also showed the formation of hepatic steatosis after HFHFD induction for 8 weeks [35]. Furthermore, the histology of the liver (Figures 3B and 3C) showed an accumulation of bile acids (cholate stasis) [16, 36], which was characterized by the appearance of enlarged hepatocytes with a size of 2–3 times larger than normal cells, become more rounded with pale cytoplasm (feathery degeneration), without any signs of accumulation of bilirubin. These histological findings were supported by serum ALP levels, which increased 2–4 times in the HFHFD and HFHFD + CCl4 groups compared to SC and SC + CCl4 groups. Theoretically, the increase in serum ALP levels occurred mainly due to an increase in the translation of alkaline phosphatase mRNA mediated by an increase in bile acid concentrations and an increase in their secretion into the circulation via canalicular leakage into the hepatic sinusoids [37]. The increase in bile acids in this study could be associated with high cholesterol levels, the precursor of bile acid synthesis, both obtained through HFD and through the promotion of cholesterol synthesis by a high-fructose diet. A previous study showed that a high-fructose diet alone could promote hepatic cholesterol synthesis [38]. Furthermore, high concentrations of circulating fatty acids both from HFD and changes in metabolism towards de novo lipogenesis (DNL) due to a high-fructose diet could activate transcription factors that regulate the expression of enzymes involved in bile acid synthesis [39, 40]. Meanwhile, this study found that serum ALT and AST levels did not increase. These results indicated that ALT and AST levels begin to rise after more pronounced steatohepatitis. Part of this study published earlier showed a significant increase in high-sensitivity C-reactive protein (hs-CRP) levels in the HFHFD and HFHFD + CCl4 groups [41]. So, the process of steatohepatitis might have already taken place in the liver of HFHFD and HHFFD + CCl4 rats, but there has not been severe hepatocyte damage, so the serum levels of the two enzymes did not increase yet.

This study also showed increased serum uric acid levels in the HFHFD and HFHFD + CCl4 groups. Hyperuricemia has long been associated with high-fat diets [42] and high-fructose diets [43]. Significant elevation of uric acid levels is associated with dyslipidemia and metabolic syndrome [44, 45]. Research in mouse models showed that adipose tissue could secrete uric acid and uric acid production increased in obesity [45]. On the other hand, a study revealed that hyperuricemia contributed to liver lipogenesis by induction of nicotinamide adenine dinucleotide phosphate (NADPH) oxidase and oxidative stress [46]. Furthermore, fructose has a significant role in hyperuricemia [47] through increased activity of ketohexokinase (KHK) on adenosine monophosphate (AMP) degradation [48, 49] and induction of increased serum lactate which then competes with uric acid for excretion by the kidney [50]. The last circumstances might contribute to the underlying histologic abnormalities of the kidneys in the HFHFD group.

In addition to the liver, the histopathological findings were most prominent in the kidney (Figure 3 C, D). By assessing the presence of hydropic degeneration, hemorrhagic, glomerular proliferation, and infiltration of inflammatory cells, the HFHFD group showed moderate kidney lesions while the SC group had minimal lesions. Lipids have been known for inducing glomerular lesions. Research on animal models fed a high-cholesterol diet and experiencing hyperlipidemia showed progressive glomerulosclerosis and renal lesions [51]. Recent studies found that a high-fat diet causes oxidative stress, characterized by mitochondrial dysfunction and increased ROS production, leading to molecular reactions towards renal cell lesions [52]. Other studies also found that consuming a high-fat diet led to fat accumulation, increased inflammatory cytokines, induction of glomerular retraction, and renal dysfunction [53]. A high-fructose diet also affected renal health through its metabolic consequences of DNL and increased production of uric acid, and induced renal hypertrophy [54, 55]. Part of this study that was published earlier found increased levels of cystatin C, a marker of renal function, in the HFHFD and HFHFD + CCl4 groups [56].

The histopathological features of the pancreas (Figure 3 A,B) showed a slight vacuolization, which indicated that fats began to accumulate in the pancreatic tissues. The spleen histology (Figure 3 E-H) showed a thickening of the Billroth cord and increased inflammatory cells. These results are in line with a previous study showing histological changes in the spleen after a high-calorie diet [17]. The effect of HFHFD on immune system function is significant to be explored further, considering that inflammatory factors play an essential role in determining the course of NAFLD and the metabolic syndrome related to obesity. For muscle histology (Figure 3I,J), this study found increased inflammatory cells. By referring to a previous study, this condition could be induced by an increased influx of fatty acids into the muscle [57].

This study have some limitations included 1) the idea that the HFHFD formula used in this study did not induce obesity, only weight gain as in the SC group, should be supported by comparing the weight of white adipose tissues (WAT) and brown adipose tissue (BAT) between the experimental groups so that it would be adequately explained; 2) measurement of free fatty acid levels in blood and liver would be better in elucidating the extent to which HFHFD affected metabolic homeostasis; 3) measurement of serum and liver bile acid levels were also necessary to be carried out to support the explanation of the increased serum ALP levels rather than only by observing the histological results of cholate stasis; 4) examination of the key enzymes, including fatty acid synthase, 3-hydroxy-3-methylglutarylcoenzyme A (HMG-CoA) reductase, and ketohexokinase activity, were also needed to confirm the explanation of the role of fructose in metabolic changes, including increased cholesterol synthesis, DNL, and hyperuricemia; 5) measurement of serum creatinine levels and glomerular filtration rate would be useful for supporting the results of kidney histopathology.

## 5 Conclusions

A high-fat diet with a formulation as applied in this study containing the main fatty acids of linoleic acid, stearic acid, myristic acid, and palmitoleic acid, and an additional high-fructose diet was able to induce severe hepatic steatosis with early signs of steatohepatitis accompanied by increased levels of serum ALP, triglycerides, total cholesterol, and uric acids, without promoting obesity within 8 weeks of administration. In addition, HFHFD also induced moderate lesions in the kidneys with a slight vacuolization of pancreas and a mild increase of inflammatory cells in spleen and muscles.

## Declarations

### Author contribution statement

Ika Yustisia, PhD; Delvina Tandiari: Conceived and designed the experiments; Performed the experiments; Analyzed and interpreted the data; Contributed reagents, materials, analysis tools or data; Wrote the paper.

Muhammad Husni Cangara, PhD; Firdaus Hamid, PhD; Nu'man AS Daud, PhD: Analyzed and interpreted the data.

### Funding statement

Dr. Ika Yustisia was supported by Hibah Penelitian Dasar 2021, Lembaga Penelitian dan Pengabdian kepada Masyarakat, Universitas Hasanuddin, Indonesia [915/UN4.22/PT.01.03/2021].

### Data availability statement

Data included in article/supp. material/referenced in article.

### Declaration of interests statement

The authors declare no conflict of interest.

### Additional information

No additional information is available for this paper.
